# Polymer-Functionalized Magnetic Nanoparticles: Synthesis, Characterization, and Methylene Blue Adsorption

**DOI:** 10.3390/ma11081312

**Published:** 2018-07-29

**Authors:** Xinyu Zheng, Huaili Zheng, Rui Zhao, Yongjun Sun, Qiang Sun, Shixin Zhang, Yongzhi Liu

**Affiliations:** 1 Key laboratory of the Three Gorges Reservoir Region’s Eco-Environment, State Ministry of Education, Chongqing University, Chongqing 400045, China; 20126128@cqu.edu.cn (X.Z.); zr864361353@126.com (R.Z.); sunqiang6666666@sina.com (Q.S.); 20116511@cqu.edu.cn (S.Z.); 2 College of Urban Construction, Nanjing Tech University, Nanjing 211800, China; sunyongjun@njtech.edu.cn; 3 National Centre for International Research of Low-carbon and Green Buildings, Chongqing University, Chongqing 400045, China; yongzhi18653028083@126.com

**Keywords:** magnetic nanoparticles, acrylic acid, 2-acrylamido-2-methyl-1-propanesulfonic acid, adsorption, methylene blue

## Abstract

The removal of methylene blue (MB) from wastewater has attracted global concerns. In this study, polymer-functionalized magnetic nanoparticles for MB removal, Fe_3_O_4_@SiO_2_-MPS-g-AA-AMPS (FSMAA), were successfully synthesized by grafting acrylic acid (AA) and 2-acrylamido-2-methyl-1-propanesulfonic acid (AMPS) on the surface of vinyl-modified Fe_3_O_4_@SiO_2_. With various characterization techniques, it was confirmed that the obtained FSMAA had a core–shell structure, a good magnetic property, and plenty of functional groups on its surface. MB adsorption experiments showed that the adsorption capacity of FSMAA was notably enhanced as the grafted monomer concentration and solution pH were increased. The adsorption kinetic data and isothermal data were well described by the pseudo-second-order kinetic model and the Langmuir model, respectively. The maximum adsorption capacity of FSMAA was 421.9 mg g^−1^ with grafted monomer concentration at 2.0 mol L^−1^ and solution pH at 9, much higher than those of other adsorbents stated in previous literatures. Based on XPS analysis, surface adsorption mechanism between FSMAA and MB was electrostatic interaction, hydrogen bonding, and hydrophobic interaction. Furthermore, FSMAA was effectively regenerated by acid pickling, and the remaining adsorption capacity was more than 60% after eight adsorption–regeneration cycles. All the results demonstrated the self-made FSMAA was a desirable adsorbent to remove MB from wastewater.

## 1. Introduction

With industry development, the demand of dyes widely used in textiles, artificial fibers, plastics, foodstuffs, and leathers rises year by year [1]. Dyes discharged into waterbodies can reduce the dissolved oxygen content and the light transmittance while increasing the toxicity of water, resulting in the death of aquatic organisms [2]. Moreover, as strongly toxic and carcinogenic pollutants, dyes pose a serious threat to water environmental safety and human health. In water, even minute quantities of some dyes (e.g., less than 1 ppm) are harmful and undesirable [3,4]. Methylene blue (MB) is one of the most commonly used basic dyes in industry that causes serious human health problems, including vomiting, shock, limb paralysis, tissue death, and so on when it is released into drinking water [5,6]. MB is difficult to be degraded under natural conditions due to its complex and stable aromatic molecular structure. As a result, the removal of such a dye from wastewater has attracted global concerns. According to literatures, multifarious techniques have been used for the treatment of wastewater containing MB, such as adsorption [5], photocatalytic degradation [7], Fenton-like degradation [8], membrane separation [9], electrochemical process [10], etc. Among these techniques, adsorption is thought to be a preferred method because of its low cost, simple design and operation, high efficiency, and insensitivity to poisonous substances [3,11].

Fe_3_O_4_ magnetic nanoparticles (MNPs) are currently regarded as base materials for their advantages, such as high surface area, facile synthesis, and low toxicity [12]. However, due to their poor stability in extremely acid environment (i.e., pH <2), the regeneration process (usually under acid environment) is limited, in other words, the application of Fe_3_O_4_ MNPs as adsorbents is limited. Furthermore, the surface of Fe_3_O_4_ MNPs, including surface charge, tendency of aggregation, and so on, is a limitation for their application. A frequently-used method to overcome this disadvantage is to cover Fe_3_O_4_ MNPs by an outer silica shell that acts as a protective layer and separates the magnetic core from the external environment to achieve a better stability [13]. However, the adsorption performance of Fe_3_O_4_@SiO_2_ MNPs is usually insufficient because of few functional groups on its surface. As a result, organic polymers with plenty of functional groups are generally adopted to functionalize Fe_3_O_4_@SiO_2_ MNPs. For example, Yure Ge et al. [13] used acrylamide, methylacryloxyethyltrimethyl ammonium chloride, and 2-acrylamido-2-methyl-1-propanesulfonic acid to functionalize Fe_3_O_4_@SiO_2_ MNPs. The multifunctionalized MNPs were proven to be effective for oilfield wastewater purification. Farzad Javaheri and Shadi Hassanajili [14] studied the removal of nitrate ions from aqueous solutions by poly(4-vinylpyridine)-functionalized MNPs. Kun Li et al. [15] adopted chitosan and polyacrylamide to functionalize Fe_3_O_4_@SiO_2_ MNPs. The obtained composite had a highly selective adsorption of mercury ions from water.

In this study, polymer-functionalized MNPs with core–shell structure named Fe_3_O_4_@SiO_2_-MPS-g-AA-AMPS (FSMAA) were synthesized as a novel adsorbent for the removal of MB from aqueous solutions. Various characterization techniques were used to characterize the structure, physic-chemical properties, and magnetic feature of FSMAA. The effects of grafted monomer concentration and solution pH on the adsorption performance were investigated. Adsorption kinetics, isotherms, and mechanism were also studied. The stability and regeneration ability of FSMAA were discussed to demonstrate its application prospect. 

## 2. Materials and Methods

### 2.1. Materials

Triiron tetraoxide (Fe_3_O_4_, 50 nm) was obtained from Micxy Chemical Co., Ltd. (Chengdu, China). Ammonia solution (25–28% NH_3_ in H_2_O), anhydrous ethanol (99.7% purity), hydrochloric acid (HCl), and sodium hydroxide (NaOH) were purchased from Chongqing Chuandong Chemical Co., Ltd. (Chongqing, China). Ammonium persulfate, methylene blue (MB), tetraethyl orthosilicate (TEOS), and acrylic acid (AA, 99.5% purity) were obtained from Chengdu Kelong Chemical Reagent Co., Ltd. (Chengdu, China). 2-acrylamido-2-methyl-1-propanesulfonic acid (AMPS, 98% purity) and 3-(trimethoxysilyl)propyl methacrylate (MPS, 97% purity) were purchased from Aladdin Reagent Co., Ltd. (Shanghai, China). All reagents were used without further treatment and Milli-Q ultrapure water (18 M Ω cm^−1^) was used for preparing aqueous solutions.

### 2.2. Synthesis of Magnetic Adsorbent

#### 2.2.1. Synthesis of Fe_3_O_4_@SiO_2_ MNPs

Fe_3_O_4_@SiO_2_ MNPs were synthesized by Stöber method according to the previous researches [13,16]. Fe_3_O_4_ (1.2 g) was homogeneously dispersed in the mixture of anhydrous ethanol (400 mL), ultrapure water (200 mL), and ammonia solution (6 mL). Then 18 mL TEOS was slowly added and the resulting dispersion was mechanically stirred for 8 h in a water bath (25 °C). Finally, the obtained Fe_3_O_4_@SiO_2_ MNPs were separated by a magnet, washed repeatedly with anhydrous ethanol and ultrapure water, and dried in a vacuum oven at 60 °C.

#### 2.2.2. Synthesis of Fe_3_O_4_@SiO_2_-MPS MNPs

The surface-modified Fe_3_O_4_@SiO_2_ MNPs were synthesized according to a reported literature with the method being slightly changed [13]. Fe_3_O_4_@SiO_2_ MNPs were dispersed in a three-necked flask containing 200 mL anhydrous ethanol. A mixture of MPS (9 mL) and anhydrous ethanol (100 mL) was added dropwise into the flask after the dispersion was completely deoxygenated by bubbling with pure N_2_ (99.99%). Then the flask was immediately sealed and mechanically stirred for 12 h in a water bath (78 °C). Lastly, the vinyl-modified Fe_3_O_4_@SiO_2_ MNPs (Fe_3_O_4_@SiO_2_-MPS) were collected by magnetic separation, washed repeatedly with anhydrous ethanol and ultrapure water, and dried subsequently in a vacuum oven at 60 °C.

#### 2.2.3. Synthesis of FSMAA MNPs

FSMAA MNPs were synthesized by one-pot free radical polymerization. Fe_3_O_4_@SiO_2_-MPS MNPs (0.35 g) were dispersed in a three-necked flask containing 100 mL ultrapure water and sonicated for 5 min to form a homogeneous dispersion. Then, a predetermined amount of solution containing grafted monomers, AA and AMPS (i.e., the mole ratio of AA and AMPS = 3:1; the grafted monomer concentration = 0.2, 0.9, 1.2, 1.5, 2.0, 2.5 mol L^−1^), was added into the dispersion. The flask was bubbled with pure N_2_ (99.99%) for 20 min to remove oxygen absolutely. After a certain mass of initiator ammonium persulfate (i.e., the initiator concentration = 0.9‰) being added, the flask was immediately sealed and mechanically stirred for 7 h in a water bath (65 °C). Finally, the obtained magnetic adsorbent was harvested via magnetic separation, washed repeatedly with anhydrous ethanol and ultrapure water, and dried subsequently in a vacuum oven at 60 °C.

### 2.3. Characterization

The morphology analysis was carried out by transmission electron microscope (TEM, HT7700, Hitachi, Tokyo, Japan). Fourier transform infrared (FTIR) spectra were made by a spotlight 200 FTIR spectrometer (Nicolet iS5, Nicolet, Madison, WI, USA) in the wavenumber range from 4000 to 500 cm^−1^. X-ray diffraction (XRD) patterns were obtained by an X-ray diffractometer (DMAX/2C, Rigaku, Tokyo, Japan) with the graphite monochromatized Cu-Kα radiation (λ = 1.54056 Å). X-ray photoelectron spectroscopy (XPS) spectra were recorded by an XPS spectrometer (ESCALAB250Xi, Thermo Fisher Scientific, Waltham, MA, USA) with Al-Kα X-ray used as the excitation source. Magnetic hysteresis loops were determined by a vibrating sample magnetometer (VSM, VSM 7410, LakeShore, Carson, CA, USA) at room temperature. Zeta potentials were measured by a Zetasizer Nano ZS90 (Malvern Instruments Ltd., Malvern, UK). The leaching concentration of Fe in water was tested with an inductively coupled plasma-optical emission spectroscope (ICP-OES, Optima 2100DV, Perkin-Elmer Instruments, Waltham, MA, USA).

### 2.4. Adsorption Experiments

**Figure 1:**
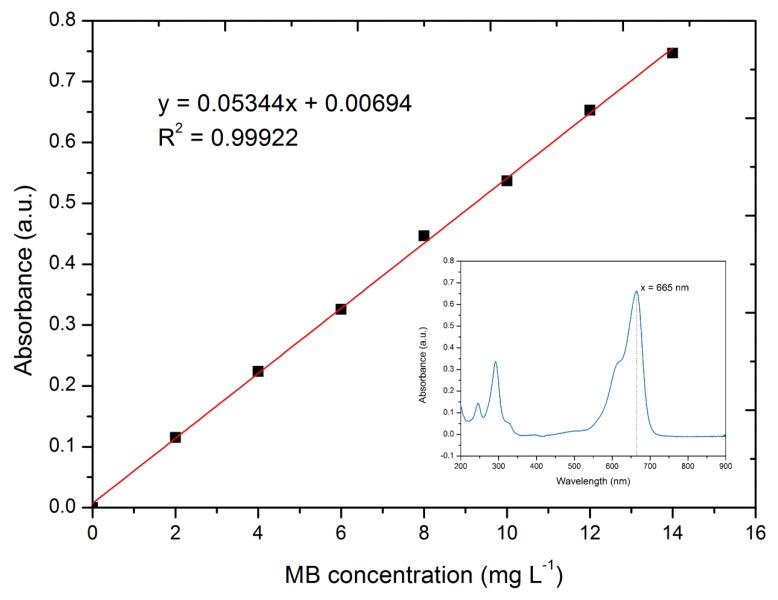
Calibration curve between MB concentration and absorbance (the insert is the UV–vis spectrum of MB in solution).

The adsorption ability of FSMAA was investigated by batch adsorption experiments with MB used as the target adsorbate. All the experiments were conducted in 50 mL conical flasks containing certain volumes of MB solutions and 1.0 g L^−1^ FSMAA. These flasks were shaken at 303 K in a constant temperature shaking bath with a shaking speed of 200 rpm. After a predetermined time, sample solutions were taken out by a transfer liquid gun under the help of an external magnet to avoid FSMAA being simultaneously taken out. The concentrations of MB in sample solutions were calculated via the calibration curve (shown in Figure 1) between MB concentration and absorbance measured by a UV-visible spectrophotometer (TU-1901, Beijing Purkinje General Instrument Co., Ltd., Beijing, China) at the maximum adsorption wavelength of 665 nm (referring to the insert of Figure 1). Each adsorption experiment was repeated three times and the final results were averaged.

#### 2.4.1. Effects of Grafted Monomer Concentration and Solution pH

The effects of grafted monomer concentration and solution pH on the adsorption performance were investigated with monomer concentrations from 0.2 to 2.5 mol L^−1^ and pH from 2.0 to 10.0, respectively. The solution pH was adjusted with 1 mol L^−1^ NaOH and/or 1 mol L^−1^ HCl solution. The initial concentration and the volume of MB solution were 500 mg L^−1^ and 10 mL, respectively, and the shaking time was 4 h. The equilibrium adsorption amount of MB (q_e_, mg g^−1^) was calculated by Equation (1).

(1) $$q_{e}=\frac{V\times \left(C_{i}-C_{e}\right)}{m}$$

where C_i_ (mg L^−1^) and C_e_ (mg L^−1^) are the initial and the equilibrium concentrations of MB, respectively, V (L) is the volume of the MB solution, and m (g) is the mass of FSMAA.

#### 2.4.2. Adsorption Kinetics

In adsorption kinetic experiments, the initial concentration, volume, and pH of MB solution were 500 mg L^−1^, 100 mL, and 9, respectively. At predetermined time intervals, 0.5 mL sample solutions were taken out by magnetic separation to analyze MB concentrations remaining in the solution. The pseudo-first-order, pseudo-second-order, and intraparticle diffusion kinetic models were adopted to analyze the adsorption data, as shown in Equations (2)–(4), respectively.

(2) $$\ln\left(q_{e}-q_{t}\right)={\mathrm{lnq}}_{e}-k_{1}t$$

(3) $$\frac{t}{q_{t}}=\frac{1}{k_{2}q_{e}^{2}}+\frac{t}{q_{e}}$$

(4) $$q_{t}=k_{i}\times t^{1/2}+C$$

(5) $$q_{t}=\frac{V\times \left(C_{i}-C_{t}\right)}{m}$$

where q_t_ (mg g^−1^) is the adsorption amount of MB at time t (min), k_1_ (min^−1^), k_2_ (g mg^−1^ min^−1^), and k_i_ (mg g^−1^ min^−1/2^) are the rate constants of pseudo-first-order model, pseudo-second-order model, and intraparticle diffusion model, respectively, and C is the parameter.

#### 2.4.3. Adsorption Isotherms

In adsorption isothermal experiments, the shaking time, volume, and pH of MB solution were 4 h, 10 mL, and 9, respectively. The initial concentration of MB solution was set from 50 mg L^−1^ to 1000 mg L^−1^. Three isothermal adsorption models, including Langmuir, Freundlich, and Dubinin–Radushkevich (D-R), shown in Equations (6)–(8), respectively, were used to analyze the adsorption data.

(6) $$\frac{C_{e}}{q_{e}}=\frac{C_{e}}{q_{m}}+\frac{1}{q_{m}\times K_{L}}$$

(7) $${\mathrm{lnq}}_{e}={\mathrm{lnK}}_{F}+\frac{1}{n}{\mathrm{lnC}}_{e}$$

(8) $${\mathrm{lnq}}_{e}={\mathrm{lnq}}_{D}-K_{D}\times {\left[\mathrm{RT}\times \ln\left(1+\frac{1}{C_{e}}\right)\right]}^{2}$$

where q_m_ (mg g^−1^) and q_D_ (mg g^−1^) are the maximum adsorption amounts of MB in the Langmuir model and in the D-R model, respectively; K_L_ (L mg^−1^), K_F_, and K_D_ (mol^2^ kJ^−2^) are the model constants of the Langmuir model, Freundlich model, and D-R model, respectively; n is the other model constant of the Freundlich model; R (J mol^−1^ K^−1^) is the gas constant; and T (K) is the thermodynamic temperature.

### 2.5. Stability and Regeneration Experiments

In stability experiments, 10 mg FSMAA was dispersed separately in 11 conical flasks, each containing 10 mL water with pH values ranged from 0 to 10. The leaching concentrations of Fe in water were tested with ICP-OES after these flasks were shaken for 12 h at 303 K in a constant temperature shaking bath. In regeneration experiments, 1 mol L^−1^, 0.1 mol L^−1^, and 0.01 mol L^−1^ HCl were selected as the desorbents. Firstly, 80 mg FSMAA was added into 80 mL MB solution, and after the adsorption was completed, the MB-loaded adsorbent (FSMAA-MB) was taken out by magnetic separation. Secondly, FSMAA-MB was dispersed in 80 mL desorbent. The regeneration reaction was carried out for 12 h at 303 K in a constant temperature shaking bath. Lastly, the regenerative FSMAA was used for the next adsorption–regeneration cycle. The adsorption–regeneration experiments were repeated for eight cycles in total.

## 3. Results and Discussion

### 3.1. Synthesis and Characterization

**Scheme 1:**
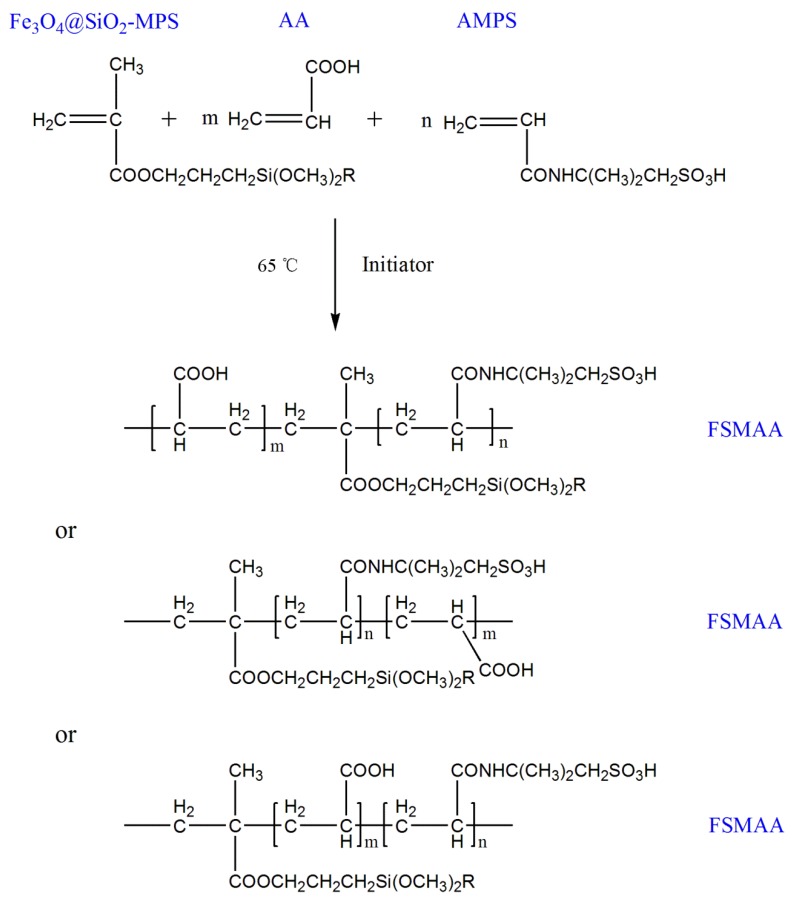
Scheme of grafting polymerization (R represents Fe_3_O_4_@SiO_2_).

**Figure 2:**
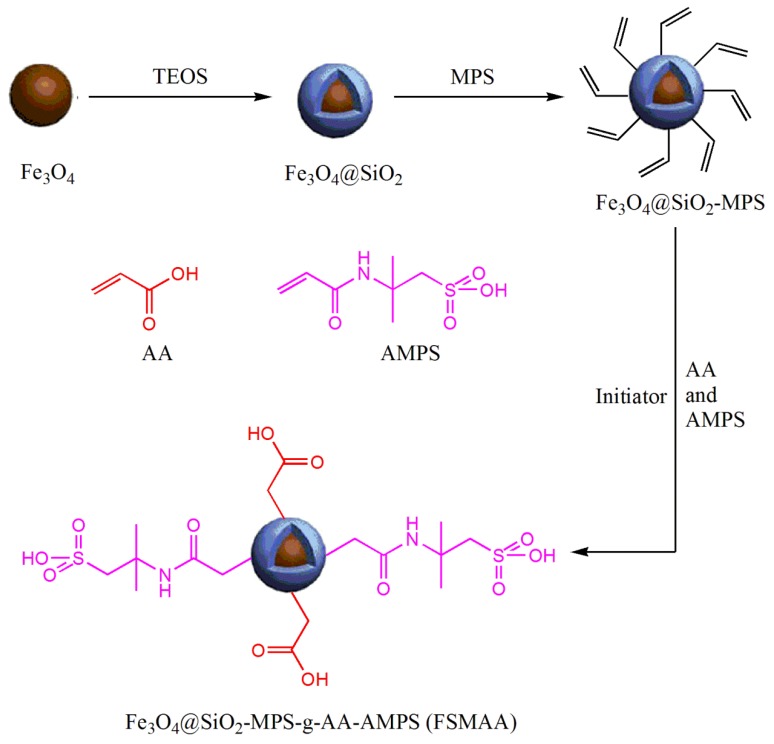
Schematic diagram on synthesis of FSMAA.

The magnetic adsorbent FSMAA with a core–shell structure was synthesized by three steps: (1) covering the Fe_3_O_4_ magnetic core with a silica shell by Stöber method; (2) modifying the silica shell with silane coupling agent MPS; and (3) obtaining the polymer-functionalized MNPs by grafting AA and AMPS on the surface of Fe_3_O_4_@SiO_2_-MPS. The grafting reaction followed the general reaction rules of free radical polymerization and it could be divided into several stages, including chain initiation, chain growth, chain termination, and a certain degree of chain transfer. The grafting route is depicted in Scheme 1 and the whole synthesis process is illustrated in Figure 2.

**Figure 3:**
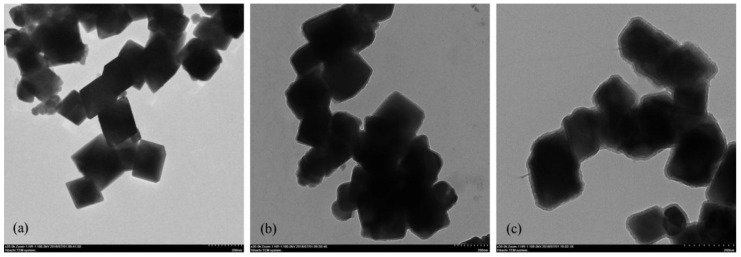
TEM images of (**a**) Fe_3_O_4_, (**b**) Fe_3_O_4_@SiO_2_, and (**c**) FSMAA (the scale bars were 200 nm for **a**, **b**, and **c**).

The core–shell structure of FSMAA was demonstrated by TEM, as seen in Figure 3. For FSMAA and Fe_3_O_4_@SiO_2_, there were bright layers of shell with main thicknesses of 14.3 nm and 10.5 nm, respectively, while for Fe_3_O_4_, no layer was observed. This phenomenon indicated the successful synthesis of polymer and SiO_2_. Fe_3_O_4_, Fe_3_O_4_@SiO_2_, and FSMAA were almost spherical and had a certain degree of adhering owing to their nanometer size and high reactivity [17].

**Figure 4:**
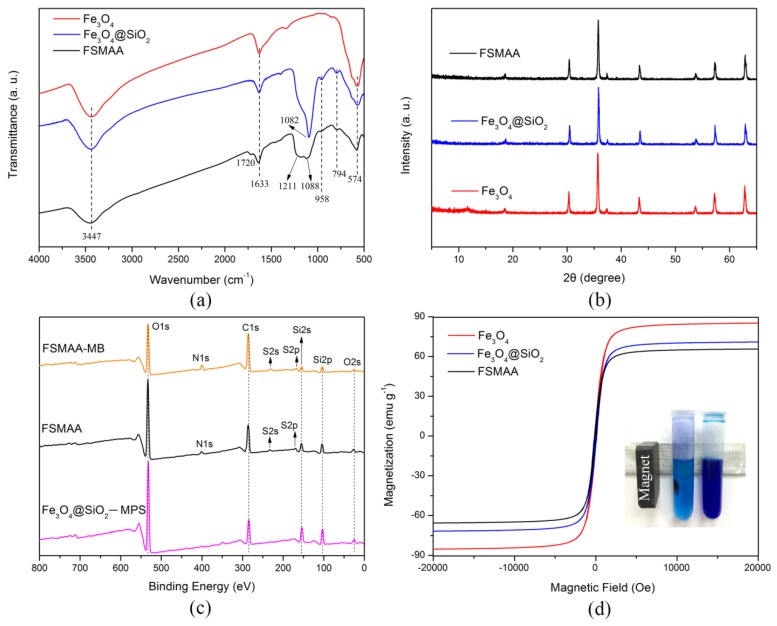
Characterization results of (**a**) FTIR spectra; (**b**) XRD patterns; (**c**) XPS full scanned spectra; (**d**) magnetic hysteresis loops (the insert is the image of MB solutions before and after adsorption; the adsorbent dosage, initial concentration, and pH are 1.0 g L^−1^, 200 mg L^−1^, and 9.0, respectively).

Figure 4a shows the FTIR spectra of Fe_3_O_4_, Fe_3_O_4_@SiO_2_, and FSMAA. The adsorption peaks at 574 cm^−1^, 1633 cm^−1^, and 3447 cm^−1^ appeared in all samples’ spectra and were attributed to the stretching vibration of Fe–O, –OH, and –OH bonds, respectively [18,19]. For Fe_3_O_4_@SiO_2_, the adsorption peaks at 794 cm^−1^ and 1082 cm^−1^ corresponded to the amorphous silica Si–O–Si vibration, and the peak at 958 cm^−1^ was associated with the Si–OH vibration [20,21]. The successful synthesis of SiO_2_ shell was demonstrated by these above peaks. For FSMAA, the adsorption peaks at 1211 cm^−1^ and 1088 cm^−1^ were the asymmetric and symmetric bands of SO_2_ in –SO_3_H, respectively [13], and the adsorption peak at 1720 cm^−1^ was assigned to –COOH bond [22]. Notably, the Si–O–Si bond at 1082 cm^−1^ was covered by the symmetric band of SO_2_ in –SO_3_H at 1088 cm^−1^. The appearance of –COOH and –SO_3_H in FSMAA indicated that the organic polymer was successfully grafted on the surface of Fe_3_O_4_@SiO_2_-MPS.

The XRD patterns were studied to investigate the crystal structures of Fe_3_O_4_, Fe_3_O_4_@SiO_2_, and FSMAA, as shown in Figure 4b. The fact that the characteristic peaks located at 30.4°, 35.7°, 43.3°, 53.7°, 57.2°, and 62.8° respectively assigned to (220), (311), (400), (422), (511), and (440) planes of Fe_3_O_4_ (JCPDS card No. 19-0629) were maintained in Fe_3_O_4_@SiO_2_ and FSMAA indicated that the coating process did not change the crystal phases of Fe_3_O_4_ [23]. 

The qualitative analysis of chemical elements was conducted by XPS fully scanned spectra, as shown in Figure 4c. The existence of Si in all samples indicated a successful synthesis of SiO_2_. New elements, including N and S, showing up in FSMAA and FSMAA-MB illustrated the successful grafting of AMPS. Since no peak corresponding to Fe was observed in all samples’ spectra, the coating of SiO_2_ was confirmed. And as there were lower intensity peaks corresponding to Si in the spectra of FSMAA and FSMAA-MB, the existence of AA and AMPS could be inferred.

Figure 4d shows the magnetic hysteresis loops of Fe_3_O_4_, Fe_3_O_4_@SiO_2_, and FSMAA. The obtained saturation magnetization values at room temperature were 84.94, 71.33, and 65.31 emu g^−1^ for Fe_3_O_4_, Fe_3_O_4_@SiO_2_, and FSMAA, respectively. Although the increase of nonmagnetic substances, including SiO_2_, AA, and AMPS, caused the decrease of saturation magnetization, the final product FSMAA still remained a high saturation magnetization that could significantly accelerate separation, as shown in the insert of Figure 4d. 

### 3.2. Adsorption of MB

#### 3.2.1. Effect of Grafted Monomer Concentration on Adsorption

**Figure 5:**
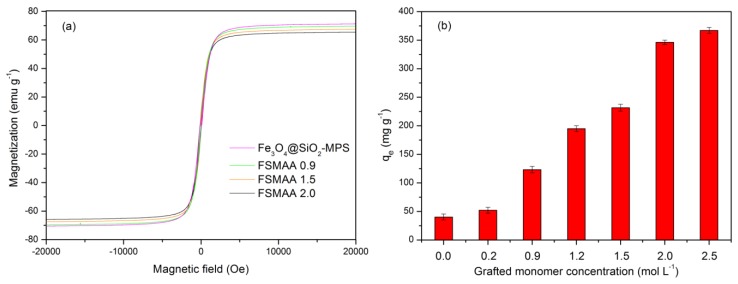
Effect of grafted monomer concentration on (**a**) magnetization and (**b**) adsorption (solution pH = 9.0).

As illustrated in Figure 5a, the saturation magnetization of synthetic products decreased from 70.90 emu g^−1^ to 65.31 emu g^−1^ with the increase of grafted monomer concentration from 0.0 mol L^−1^ to 2.0 mol L^−1^, demonstrating more polymers were grafted onto MNPs. Figure 5b shows that the grafting of AA and AMPS on the surface of Fe_3_O_4_@SiO_2_-MPS has a good effect on MB adsorption. Since there were more and more polymers with abundant functional groups being introduced on the surface of Fe_3_O_4_@SiO_2_-MPS with the increase of grafted monomer concentration from 0.0 mol L^−1^ to 2.0 mol L^−1^, the equilibrium adsorption amount significantly increased from 40.2 mg g^−1^ to 346.2 mg g^−1^. However, as the monomer concentration further increased to 2.5 mol L^−1^, there was only a little increase in equilibrium adsorption amount. This phenomenon was due to the possibility that the grafted monomer concentration could largely affect polymerization. A higher monomer concentration contributed to generating more monomer free radicals and to accelerating the polymerization. However, a too high polymerization speed would lead to chain transfer or chain termination, and stop the polymerization [24]. Therefore, the optimized monomer concentration was determined to be 2.0 mol L^−1^. In the following adsorption experiments, FSMAA MNPs with grafted monomer concentrations of 0.9 mol L^−1^, 1.5 mol L^−1^, and 2.0 mol L^−1^ (recorded as FSMAA 0.9, FSMAA 1.5, and FSMAA 2.0, respectively) were used as the adsorbents.

#### 3.2.2. Effect of Solution pH on Adsorption

**Figure 6:**
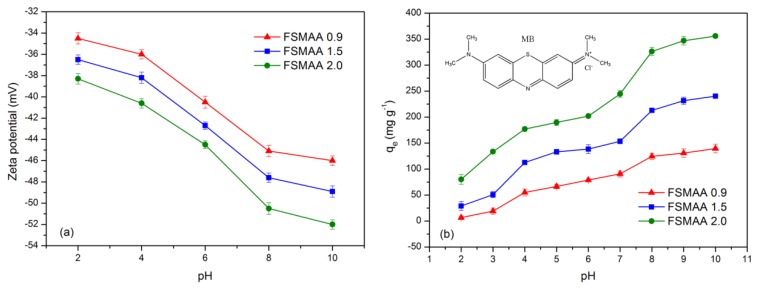
(**a**) Zeta potentials/pH profiles of FSMAA 0.9, FSMAA 1.5, and FSMAA 2.0 (electrolyte concentration = 0.7 g L^−1^); (**b**) effect of solution pH on adsorption (the insert is the image of MB’s chemical structure).

The solution pH strongly affected not only the zeta potentials on the surface of the adsorbent, but also the existence forms of the adsorbate. Since basic dyes were unstable under high pH (generally >10) environment, the solution pH value between 2 and 10 was selected to investigate its effect on MB adsorption [25]. Because of the deprotonation of –SO_3_H and –COOH, the zeta potentials on the surface of FSMAA declined with the increase of pH, as shown in Figure 6a. Among these three adsorbents, the zeta potentials of FSMAA 2.0 were the minimum in the tested pH range owing to the fact that more –SO_3_H and –COOH were introduced to its surface. Corresponding to the results obtained in zeta potentials experiments, the equilibrium adsorption amount of FSMAA significantly increased with the increase of pH and it reached the maximum when FSMAA 2.0 was used, as shown in Figure 6b. A lower pH environment weakened the electrostatic interaction between adsorbents and dyes, and it had abundant protons to compete for adsorption sites with dyes, leading to an unsatisfactory adsorption performance [26,27]. These phenomena suggested that electrostatic interaction was the main mechanism of FSMAA to remove MB from wastewater. 

#### 3.2.3. Adsorption Kinetics

**Figure 7:**
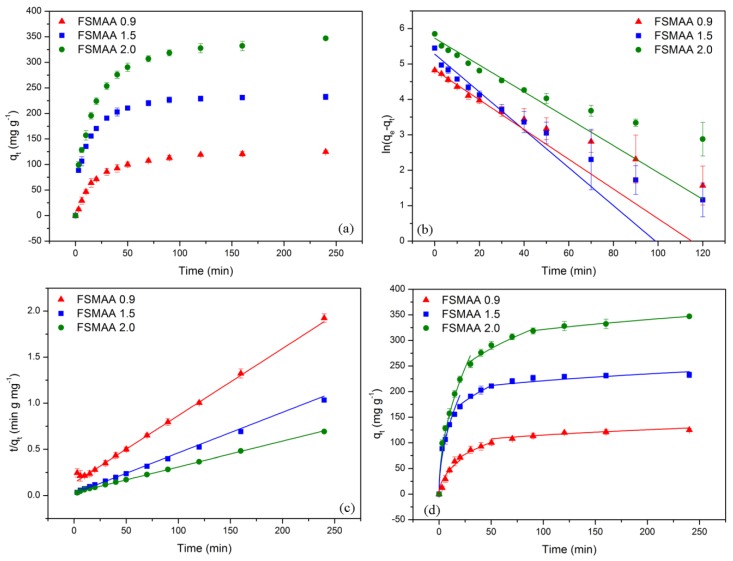
(**a**) Adsorption kinetics of MB adsorbed onto FSMAA; (**b**) fitting curves of pseudo-first-order kinetic model; (**c**) fitting curves of pseudo-second-order kinetic model; (**d**) fitting curves of intraparticle diffusion model.

In order to understand the adsorption mechanism and speed control step, adsorption kinetics was studied by using FSMAA 0.9, FSMAA 1.5, and FSMAA 2.0. Since abundant active sites on the surface of FSMAA available for adsorption were gradually occupied by MB with the increase of time, the adsorption of MB was very fast in the initial stage, then increased slightly and finally reached a plateau, as illustrated in Figure 7a; this phenomenon was also observed in other adsorption experiments [28]. Pseudo-first-order and pseudo-second-order kinetic models were used to analyze the kinetic data, as shown in Figure 7b,c. Correlation coefficients R^2^ and reduced Chi-Sqr were adopted to evaluate these two models; a higher R^2^ and a lower reduced Chi-Sqr indicated a stronger degree of curve fitting [29]. Table 1 reveals that the pseudo-second-order kinetic model had higher values of R^2^ and lower values of reduced Chi-Sqr than the pseudo-first-order kinetic model. Furthermore, the equilibrium adsorption amounts of FSMAA calculated by the pseudo-second-order kinetic model were closer to the experimental values than those calculated by the pseudo-first-order kinetic model. Hence, the adsorption of MB onto FSMAA was better explained with the pseudo-second-order kinetic model. When the main mechanism of dye removal was electrostatic interaction, a similar result was also obtained in other dye adsorption kinetic researches [30,31,32].

To further investigate whether intraparticle diffusion was a speed control step in MB adsorption, intraparticle diffusion model was also adopted to analyze the kinetic data. As shown in Figure 7d and Table 1, all the fitting curves were composed of three parts and the rate constant (k_i_) of each part had a character of k_i,1_ > k_i,2_ > k_i,3_. Consequently, the adsorption process could be divided into three stages [33,34]. The first was immediate diffusion stage. A large number of MB molecules were adsorbed immediately from aqueous solution by functional groups grafted on the surface of FSMAA. The fact that the rate constant of FSMAA 2.0 was higher than those of FSMAA 1.5 and FSMAA 0.9 indicated more functional groups were introduced to the surface of adsorbent with the increase of grafted monomer concentration. The second was intraparticle diffusion stage. A small number of MB molecules occupying the adsorption sites on the surface of FSMAA diffused to the inner layer of FSMAA and were adsorbed in it. The rate constants in this stage were much lower than those in the first one. The last was equilibrium stage. The rate constants were close to zero. All the adsorbents achieved adsorption equilibrium after 90 min. These results indicated that the surface adsorption was a rate-determining step and the intraparticle diffusion was a rate-influencing step in the adsorption of MB.

#### 3.2.4. Adsorption Isotherms

**Figure 8:**
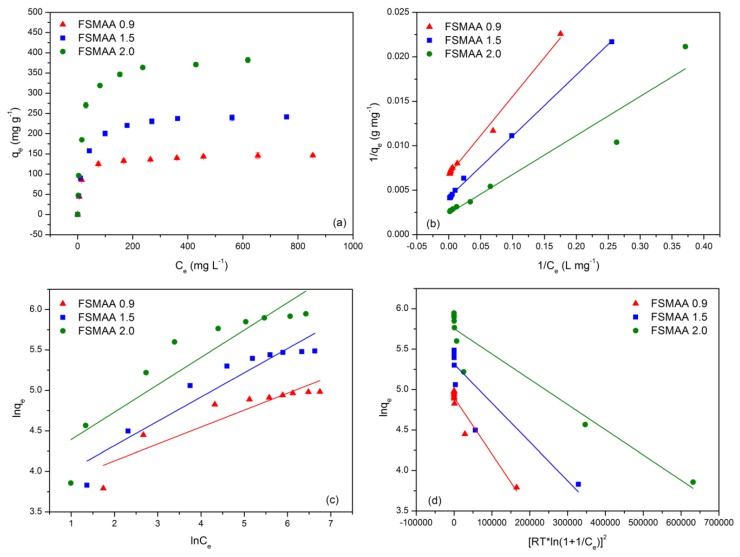
(**a**) Adsorption isotherms of MB adsorbed onto FSMAA; (**b**) fitting curves of Langmuir model; (**c**) fitting curves of Freundlich model; (**d**) fitting curves of D-R model.

The adsorption isotherms played an important role in understanding the interaction between adsorbents and adsorbates at specific temperatures and in estimating the adsorption mechanism. Three isothermal adsorption models were used to describe the adsorption process and the isothermal parameters of each model were calculated, as shown in Figure 8 and Table 2, respectively. The correlation coefficients R^2^ of the Langmuir model (≥0.929) were all higher than those of the Freundlich model (≤0.884) and the D-R model (≤0.919). Additionally, the reduced Chi-Sqr of the Langmuir model (2.479 × 10^−7^, 5.762 × 10^−8^, and 2.704 × 10^−6^ for FSMAA 0.9, FSMAA 1.5, and FSMAA 2.0, respectively) were much lower than those of the Freundlich model (0.028, 0.038, and 0.097 for FSMAA 0.9, FSMAA 1.5, and FSMAA 2.0, respectively) and the D-R model (0.013, 0.067, and 0.048 for FSMAA 0.9, FSMAA 1.5, and FSMAA 2.0, respectively). These phenomena indicated that the adsorption of MB onto FSMAA was better described by the Langmuir model. A dimensionless constant separation factor (R_L_) can be calculated by the Langmuir model, as shown in Equation (9).

(9) $$R_{L}=\frac{1}{1+K_{L}\times C_{i}}$$

**Figure 9:**
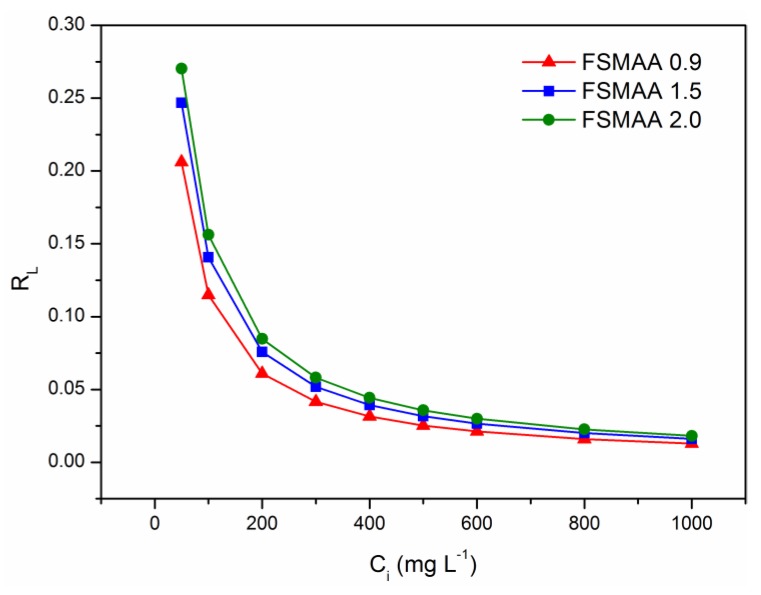
R_L_ values of MB adsorbed onto FSMAA.

0 < R_L_ < 1 implies a favorable adsorption, R_L_ > 1 means an unfavorable adsorption, R_L_ = 0 indicates an irreversible adsorption, and R_L_ = 1 means a linear adsorption [35]. As seen in Figure 9, R_L_ values of FSMAA 0.9, FSMAA 1.5, and FSMAA 2.0 calculated from the Langmuir model were all smaller than 0.30, demonstrating the adsorption of MB onto FSMAA was favorable. Furthermore, it was noted that R_L_ values decreased as the initial concentrations of MB were increased; this indicated a higher MB concentration was beneficial to adsorption.

Besides, adsorption free energy (E_a_) was calculated according to the D-R model, as shown in Equation (10).

(10) $$E_{a}={\left(2K_{D}\right)}^{-1/2}$$

E_a_ < 8 kJ mol^−1^ implies a physical adsorption, 8 kJ mol^−1^ < E_a_ < 16 kJ mol^−1^ means the adsorption mechanism is ion exchange, and E_a_ > 16 kJ mol^−1^ indicates a chemical adsorption [35]. In this work, E_a_ values of FSMAA 0.9, FSMAA 1.5, and FSMAA 2.0 were 0.27, 0.32, and 0.40, respectively.

As calculated by the Langmuir model, the maximum adsorption capacities of MB adsorbed onto FSMAA 0.9, FSMAA 1.5, and FSMAA 2.0 were 147.9 mg g^−1^, 238.1 mg g^−1^, and 421.9 mg g^−1^, respectively. Table 3 lists the maximum adsorption capacities of FSMAA 2.0 and other adsorbents for MB. As can be seen, the adsorption capacity obtained in this study was much higher than those stated in previous literatures. This conclusion showed that self-made FSMAA had good potentiality in MB removal.

#### 3.2.5. Adsorption Mechanism

**Figure 10:**
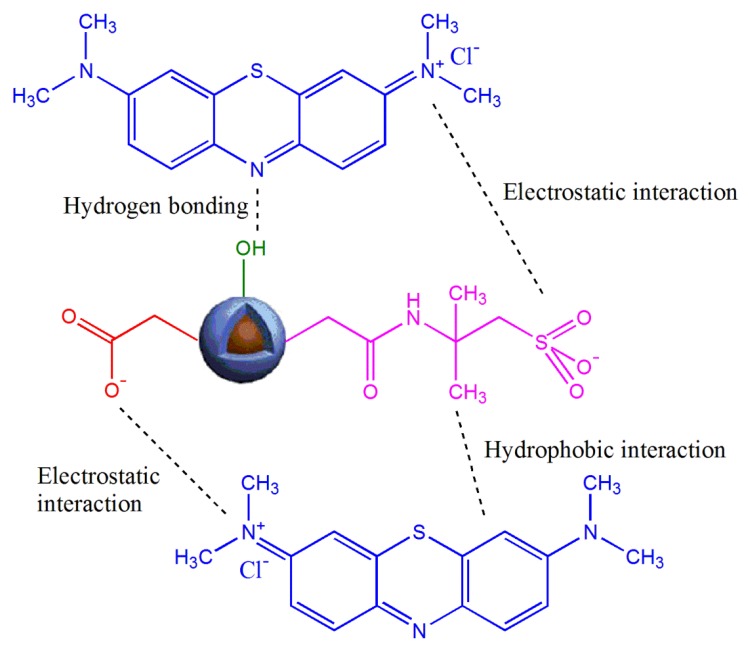
Schematic illustration of MB adsorbed onto FSMAA.

Usually if an adsorption process was better described by the pseudo-second-order kinetic model, it was inferred to be a chemical one. However, as discussed in Section 3.2.4, the fact that E_a_ calculated from the D-R model was lower than 8 kJ mol^−1^ implied the adsorption process should be a physical one. Hence, the adsorption mechanism in this study could not be simply classified as a chemical reaction or physical reaction rather, it was thought to be a combined one. Based on the results obtained in Section 3.2.3, the adsorption process was composed of surface adsorption, intraparticle diffusion adsorption, and equilibrium. Surface adsorption was the rate-determining step achieved by three types of interactions: (1) electrostatic interaction; (2) hydrogen bonding; and (3) hydrophobic interaction. Electrostatic interaction occurred between deprotonated groups (i.e., –COO^−^ and –SO_3_^−^) on the surface of FSMAA and positively charged quaternary ammonium groups in MB. Hydrogen bonding referred to the interaction between –OH, –NH on the surface of FSMAA, and amine in MB [35,43]. Hydrophobic interaction was a tendency of nonpolar groups to associate in aqueous solution. Because of the aliphatic branches on its pendant groups, the grafted AMPS had a hydrophobic character and could react with benzene rings in MB through hydrophobic interaction [44,45]. The mechanism between MB and FSMAA in surface adsorption is schematically illustrated in Figure 10.

**Figure 11:**
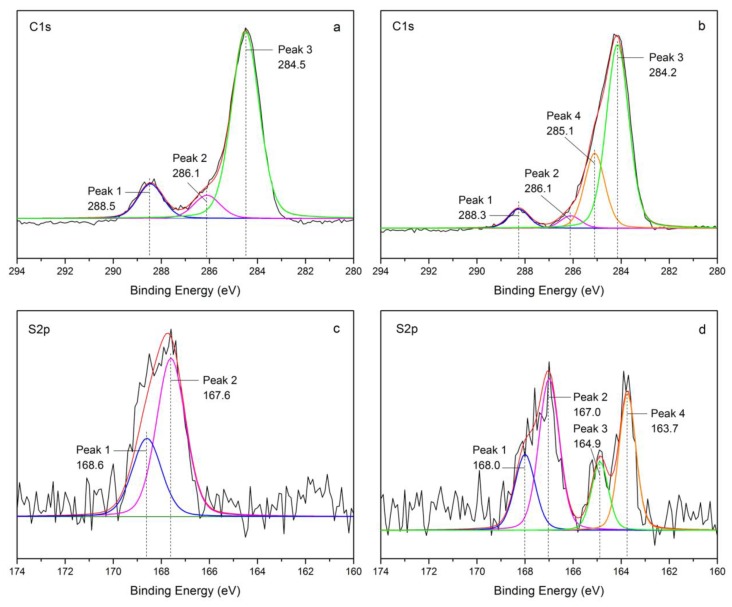
C1s and S2p XPS spectra of (**a**,**c**) FSMAA; (**b**,**d**) FSMAA-MB.

Further evidence to support the above mechanism between MB and FSMAA was provided by XPS analysis. C1s and S2p XPS spectra of FSMAA before and after adsorption are shown in Figure 11, and the assignment, binding energy, and relative area percentage of peaks are summarized in Table 4. For the C1s spectra, three peaks at about 288 eV, 286 eV, and 284 eV corresponded to C=O, C–OH/C–N/C–S, and C–H/C–C, respectively. For the S2p spectra, peaks for S2p_3/2_ at 167.0–167.6 eV and for S2p_1/2_ at 168.0–168.6 eV were assigned to S=O. The relative area percentages of C=O and S=O decreased after adsorption, suggesting carboxyl and sulfonic acid groups were involved in the adsorption of MB onto FSMAA. The new peak at 285.1 eV appearing in C1s spectrum after adsorption was attributed to the π bond formed by the phenylalkane in adsorbed MB [46]. The new peaks at 163.7 eV and 164.9 eV for S2p_3/2_ and S2p_1/2_, respectively, in S2p spectrum after adsorption were also attributed to the adsorbed MB. The change of relative area percentage of each peak and the appearance of new peaks indicated that the functional groups on the surface of FSMAA reacted with MB through electrostatic interaction, hydrogen bonding, and hydrophobic interaction.

### 3.3. Stability and Regeneration

**Figure 12:**
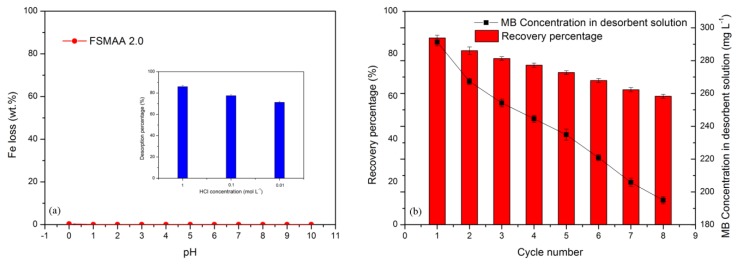
(**a**) Leaching of Fe from FSMAA in different pH solutions (the insert is the image of desorption percentages obtained by different desorbents); (**b**) recovery percentage of FSMAA and MB concentration in desorbent solution after every regeneration experiment in eight adsorption–regeneration cycles (grafted monomer concentration = 2.0 mol L^−1^).

The adsorbent’s stability and regeneration ability were important indexes in practical application. The stability of FSMAA in waters with different pH values was studied by measuring the leaching concentration of Fe. As illustrated in Figure 12a, the leaching of Fe was inhibited even in extremely acid environment (i.e., pH = 0 or 1) due to the protection of the outer polymer shell. This phenomenon demonstrated FSMAA had a good stability.

As discussed in Section 3.2.2, the adsorption performance in acid environment was unsatisfactory. It was implied that acid pickling could be a feasible way to regenerate the adsorbent [47]. Hence, 1 mol L^−1^, 0.1 mol L^−1^, and 0.01 mol L^−1^ HCl were adopted as the desorbents and the desorption performance is shown in the insert of Figure 12a. The desorption percentages for 1 mol L^−1^, 0.1 mol L^−1^, and 0.01 mol L^−1^ HCl were 85.9%, 77.5%, and 71.2%, respectively, so 1 mol L^−1^ HCl was chosen as the desorbent for the adsorption–regeneration experiments. As seen in Figure 12b, the recovery percentage of FSMAA and the MB concentration in desorbent solution after every regeneration experiment declined with the increase of cycle number. This was probably caused by the decrease of adsorbent quantity and the incomplete desorption of MB adsorbed at the bottom of grafted polymer chains. Nevertheless, the remaining adsorption capacity was more than 60% after eight adsorption–regeneration cycles, indicating FSMAA had a good regeneration ability.

## 4. Conclusions

Polymer-functionalized MNPs named FSMAA were successfully synthesized by grafting AA and AMPS on the surface of Fe_3_O_4_@SiO_2_-MPS through one-pot free radical polymerization. Various characterization techniques, including TEM, FTIR, XRD, XPS, and VSM, had been used and it was confirmed that the obtained FSMAA had a core–shell structure, a good magnetic property, and plenty of functional groups on its surface. MB adsorption experiments showed that the adsorption capacity of FSMAA increased with the increase of grafted monomer concentration and solution pH. The experimental kinetic data were well described by the pseudo-second-order kinetic model and the adsorption equilibrium was achieved after 90 min. Besides, the intraparticle diffusion model proved the adsorption process contained three stages. The experimental isothermal data were better explained by the Langmuir model and the maximum adsorption capacity of FSMAA 2.0 calculated from this model was 421.9 mg g^−1^, much higher than those of other adsorbents stated in previous literatures. The excellent adsorption performance was attributed to the electrostatic interaction, hydrogen bonding, and hydrophobic interaction. Furthermore, FSMAA exhibited a good stability and regeneration ability; the remaining adsorption capacity was more than 60% after eight adsorption–regeneration cycles. All the above mentioned indicated FSMAA’s potentiality of practical application in the removal of MB from wastewater. Our future work will be more concentrated on the mechanism analysis (for example, the grafting reaction studies and thermodynamic studies) and the further optimization of other synthesis conditions (for example, the amounts of added TEOS and MPS).

## Figures and Tables

**Table 1:** Kinetic parameters for MB adsorption onto FSMAA (*n* = 3, Mean ± SD).

Monomer Concentration (mol L^−1^)	q_e,exp_ (mg g^−1^)	Pseudo-First-Order Model				Pseudo-Second-Order Model				Intraparticle Diffusion Model		
		q_e1,cal_ (mg g^−1^)	k_1_ × 10^2^ (min^−1^)	R^2^	Reduced Chi-Sqr	q_e2,cal_ (mg g^−1^)	k_2_ × 10^4^ (g mg^−1^ min^−1^)	R^2^	Reduced Chi-Sqr	k_i,1_	k_i,2_	k_i,3_
										(mg g^−1^ min^−1/2^)		
0.9	124.8 ± 3.0	125.0 ± 2.6	4.20 ± 0.32	0.939	4.642	137.4 ± 1.5	3.84 ± 0.16	0.998	0.500	14.82 ± 1.36	11.20 ± 0.60	2.50 ± 0.70
1.5	232.3 ± 5.5	195.7 ± 13.2	5.34 ± 0.72	0.832	14.218	227.3 ± 4.0	8.83 ± 0.51	0.996	8.888	43.10 ± 2.86	14.44 ± 1.58	3.22 ± 0.60
2.0	346.8 ± 3.0	307.2 ± 14.8	3.79 ± 0.50	0.837	52.516	358.4 ± 4.7	2.56 ± 0.15	0.998	2.691	49.30 ± 1.19	15.69 ± 1.64	4.65 ± 0.21

**Table 2:** Isothermal parameters for MB adsorption onto FSMAA (*n* = 3, Mean ± SD).

Monomer Concentration (mol L^−1^)	Langmuir Model				Freundlich Model				D-R Model			
	q_m_ (mg g^−1^)	K_L_ (L mg^−1^)	R^2^	Reduced Chi-Sqr	n	K_F_	R^2^	Reduced Chi-Sqr	q_D_ (mg g^−1^)	K_D_ (mol^2^ kJ^−2^)	R^2^	Reduced Chi-Sqr
0.9	147.9 ± 4.2	0.077 ± 0.003	0.991	2.479 × 10^−^^7^	4.79 ± 0.81	40.95 ± 7.36	0.819	0.028	134.3 ± 5.5	6.97 ± 0.73	0.919	0.013
1.5	238.1 ± 5.2	0.061 ± 0.001	0.998	5.762 × 10^−^^8^	3.34 ± 0.43	41.28 ± 7.76	0.884	0.038	203.2 ± 19.0	4.80 ± 0.84	0.798	0.067
2.0	421.9 ± 19.4	0.054 ± 0.005	0.929	2.704 × 10^−^^6^	2.96 ± 0.50	57.73 ± 14.19	0.819	0.097	314.9 ± 26.0	3.12 ± 0.34	0.911	0.048

**Table 3:** The maximum adsorption capacities of FSMAA 2.0 and other adsorbents for MB.

Adsorbent	pH	Temperature (K)	Equilibrium Time (min)	Adsorption Capacity (mg g^−1^)	Reference
SNCM	6.0	323	30	20.0	[36]
SW-ZnO-PANI	7.0	305	100	20.6	[37]
GO-CS-Fe_3_O_4_	5.3	303	60	95.2	[38]
Fe_3_O_4_/SiO_2_-GO	N.A. ^a^	333	70	111.1	[39]
Fe_3_O_4_@C NPs	6.0	298	180	117.0	[40]
GO-Fe_3_O_4_	N.A. ^a^	298	30	167.2	[41]
CS-Glu-MCMs	7.0	293	5	182.5	[22]
MG-ILs-OH	12.0	303	60	243.3	[42]
FSMAA 2.0	9.0	303	90	421.9	This study

^a^: not avaliable.

**Table 4:** Normalized peak areas and assignments of XPS spectra.

Sample		Peak for C1s Spectra				Peak for S2p Spectra			
		Peak 1	Peak 2	Peak 3	Peak 4	Peak 1	Peak 2	Peak 3	Peak 4
FSMAA	BE (eV)	288.5	286.1	284.5	—	168.6	167.6	—	—
	RAP (%)	13.04	9.26	77.70	—	33.33	66.67	—	—
FSMAA-MB	BE (eV)	288.3	286.1	284.2	285.1	168.0	167.0	164.9	163.7
	RAP (%)	6.23	4.04	65.45	24.28	19.14	38.29	14.19	28.38
Assignment		C=O	C–OH/C–N/C–S	C–H/C–C	phenylalk-ane	S=O		sulfoether/S–H	

BE: binding energy; RAP: relative area percentage.
